# Development and Validation of a Questionnaire to Evaluate Workplace Violence in Healthcare Settings

**DOI:** 10.7759/cureus.19959

**Published:** 2021-11-28

**Authors:** Archana Kumari, Amandeep Singh, Piyush Ranjan, Siddharth Sarkar, Tanveer Kaur, Ashish D Upadhyay, Kirti Verma, Vignan Kappagantu, Ajay Mohan, Upendra Baitha

**Affiliations:** 1 Obstetrics and Gynaecology, All India Institute of Medical Sciences, New Delhi, IND; 2 Internal Medicine, All India Institute of Medical Sciences, New Delhi, IND; 3 Psychiatry, National Drug Dependence Treatment Centre, All India Institute of Medical Sciences, New Delhi, IND; 4 Medicine, All India Institute of Medical Sciences, New Delhi, IND; 5 Biostatistics, All India Institute of Medical Sciences, New Delhi, IND; 6 Obstetrics and Gynecology, Vardhaman Mahavir Medical College & Safdarjung Hospital, New Delhi, IND; 7 Emergency Medicine, All India Institute of Medical Sciences, New Delhi, IND; 8 Surgery, All India Institute of Medical Sciences, New Delhi, IND

**Keywords:** violence against doctors, violence prevention, verbal violence, environmental and occupational health, impact of violence, mitigation strategies, healthcare setting, workplace violence

## Abstract

Introduction

This study aims to develop and validate a questionnaire to assess workplace violence (WPV) domains in the healthcare setting.

Methods

* *The study used a mixed-method design. In Phase 1, qualitative methods for developing the questionnaire were employed, including literature review, focus-group discussion, expert evaluation, and pre-testing. During Phase 2, quantitative methods were employed for establishing the construct validity of the questionnaire. In Phase 1, experts from departments like emergency medicine, medicine, obstetrics and gynecology, psychiatry, trauma, anesthesia, and critical care unit participated. For Phase 2, data were collected from 213 participants; mean age (30.48±5.95) in metropolitan cities.

Results

The questionnaire consists of 37 items in five domains: (A) Forms of violence, (B) Impact of violent incidences, (C) Reporting of incidence, (D) Mitigation strategies, and (E) Risk factors. The Cronbach’s alpha value of the questionnaire is 0.86, suggesting an excellent internal consistency.

Conclusion

A reliable and valid tool for gathering information regarding WPV in the healthcare system from around the world has been developed. The tool can be used to study the elements that may contribute to violence and its consequences, which will help policymakers curate various mitigation methods to safeguard WPV victims.

## Introduction

Workplace violence (WPV) in the healthcare sector is a global concern and has become unfortunate in different parts of the world [1]. Several patients, doctors, organizational and society-related factors lead to verbal and physical forms of violence, causing a significant impact on healthcare workers’ (HCWs) social and mental well-being. The global prevalence of WPV in healthcare is estimated to be up to 80 percent [2]. The actual figure maybe even worse, as many incidents are ignored and unreported [3,4]. The need of the hour is to evolve mitigation strategies to reduce workplace violence and ensure safe working conditions for healthcare personnel, as a well-functioning healthcare sector is essential to enhance the quality of life of citizens anywhere in the world.

Researches conducted in different parts of the world have studied different aspects of the WPV in healthcare settings. Most of them have used non-validated semi-structured interview techniques, making it challenging to compare different studies [5]. Most of the available validated questionnaires are not easy to administer and suffer from the limitation of low psychometric properties. They are unable to capture various domains comprehensively as one single scale [6-10].

This study was conducted to develop and validate a comprehensive, easy-to-administered tool that could assess all relevant domains of WPV in a healthcare setting (forms of violence, impact, underreporting, mitigation strategies, and risk factors) that would help in a comprehensive evaluation.

## Materials and methods

This questionnaire was developed and validated using a standard scientific approach [11-13]. This included a thorough study of the literature, in-depth interviews, focus group discussions, expert opinions, pilot testing, and validation. The study was conducted after prior approval from the Institute Ethics Committee of The All India Institute of Medical Sciences, New Delhi, India (IEC-844/06.12.2019, RP-46/2020). All subjects involved in the research provided informed consent for the same. The process of questionnaire development is given in Table 1.

**Table 1:** Process of questionnaire development * Focus Group Discussions

Step	Nature of activity	Methods	Number of domains	Number of items at the end of step	Addition or subtraction
1	Understanding the construct	Review of literature	Nil	45	Nil
2	Development of the construct	*FGDs	5	57	12 items were added
3	Generation of items	Developed items	5	57	Nil
4	Face and content validity	Validation by experts	5	46	11 items were deleted
5	Cognitive interviewing	Pilot study	5	40	6 items were deleted
6	Construct validity	Exploratory Factor Analysis	5	37	3 items were deleted

Step 1: Literature Review 

An in-depth literature review was done by using search string (violence OR aggression) AND(doctor OR physician OR “general practitioner” OR Surgeon OR resident OR intern” OR clinicians OR “health care) AND (workplace) AND (determinants OR predictor) AND (intervention* OR strategy* OR prevent*) in PubMed and Wiley from looking for relevant studies done over the past ten years, in the English language. Five hundred fifty-three articles were screened, from which 27 relevant articles were selected. This helped identify various domains for the questionnaire like types/ forms of violence, reporting of violence, risk factors of violence, the impact of violence, and mitigation strategies under which 45 items were generated. 

Step 2: Focus group discussions (FGDs) and in-depth interviews

The investigators performed five FGDs, each session having 6-8 participants. The sessions were organized over an online platform with the doctors and other healthcare workers. As per the literature review and collaboration with an expert (clinical psychologist), the FGD guide was developed, which included open-ended questions to help participants explore their viewpoints, practices, and issues. Active involvement was encouraged, and the discussion included topics such as types/forms of violence, reporting of violence, risk factors of violence, the impact of violence, and different mitigation strategies which can be used to reduce workplace violence. Later on, five in-depth interviews were also conducted to gain more information on this issue. The resulting data were qualitatively evaluated, and new items were added to the tool as a result. The focus groups and in-depth interviews resulted in the addition of 12 items.

Step 3: Generation of items

A set of 57 questions was created based on the literature review, focus group discussions, and in-depth interviews. The survey items were prepared in an easy-to-understand format, eliminating the double negatives.

Step 4: Expert Validation

A team of eight specialists from various areas (emergency medicine, medicine, gynecology, psychiatry, clinical psychology, and nursing) validated the developed tool for critical review, content, and face validity. All elements were assessed based on their need, clarity, and relevance. The feedback made necessary adjustments to the questionnaire: 11 items were eliminated at this stage.

Step 5: Pilot Testing

Following the approach mentioned above, a questionnaire draft was created and pre-tested on 12 doctors and other health care workers. The participants remarked on the items’ need, relevance, and clarity. Six items were eliminated based on feedback and expert consultation, and necessary adjustments were made in four items to reduce ambiguity.

Validation of the questionnaire

During expert validation, the questionnaire’s content validity was examined using qualitative and quantitative approaches. An expert panel of eight participants was invited to examine the questionnaire and comment on the necessity, relevance, and clarity of items in the qualitative validity. The items were changed in response to the input. A three-point scale (-1,0,+1) was used to grade the usefulness of items. Lawshe scores were used to compute appropriate Content Validity Ratio (CVR) values. To determine the relevance, clarity and simplicity of each item, a four-point Likert scale was used. Items with a 0.7 or less Content Validity Index (CVI) were eliminated, and those between 0.7-0.79 were altered as per the expert’s opinion [14]. Expert review and pilot testing were conducted to achieve face validity from doctors and other healthcare workers belonging to different departments.

Cross-sectional survey

A cross-sectional survey was conducted to validate the questionnaire. Participants, including doctors and other healthcare staff from various departments like emergency medicine, medicine, psychiatry, nursing, obstetrics, and gynecology, were recruited during August 2021; a convenience sampling method was used to recruit the participants. After receiving written informed consent, the investigators administered the questionnaire in online and offline mode, and responses were recorded on Google forms simultaneously. 

Statistical analysis

The demographic information of the participants was analyzed using descriptive statistics. The Kaiser-Mayer-Olkin (KMO) measure determines the adequacy of the sample and values greater than 0.5 suggest that the data is suitable for factor analysis. To discover domains of the questionnaire and establish construct validity, exploratory factor analysis with principal component extraction and varimax rotation with Kaiser normalization were employed. The internal consistency was measured using Cronbach’s alpha. Its score of greater than 0.7 suggests that it has high internal consistency. IBM® SPSS® Statistics version 24.0 was used to analyze the data.

## Results

The questionnaire has 37 items, comprising of five sections viz., forms of violence, the impact of violent episodes, reporting of incidence, mitigation strategies, and risk factors related to the health care workers in the various departments and settings. The questionnaire is available in Table 2 and is free to use.

**Table 2:** Questionnaire for Workplace Violence in Healthcare Settings

Name:	
Age (In years):	Gender:
Highest Degree	Workplace Setting:
Area of workplace:	Marital status:
Department of residency/specialization/Working:	
Number of years of experience after completion of MBBS/BSc: _________________	
Section A- Forms of Violence: This domain intends to assess the frequency of various forms of violence experienced by healthcare workers in healthcare settings. Mark the most appropriate option.	
A1: How often do you experience verbal altercations (e.g., threats, abuse, exaggerated arguments, offensive comments etc.) at your workplace?	
Nearly daily	
About once a week	
About once a month	
About once every six months	
About once a year or less	
A2: How often do you experience physical violence (e.g., slapping, beating, thrashing, vandalizing, attack with weapons etc.) at your workplace?	
About once in a month or more	
About once every six months	
About once a year	
Less than once a year	
Never	
Section B- Impact of incidences of Violence: This domain assesses the impact of the episodes of violence on the various aspects of an individual’s life.	
B1: On the basis of the episodes of violence at my workplace, I have developed the following feelings:	
It did not/doesn’t affect me at all	
I feel/felt that motivation/efficiency reduced at my work	
I feel/felt like changing my workplace	
I feel/felt like opting for an alternate career	
I feel/felt like not working at all	
I have/had self-harm/suicidal ideations	
Following are the statements regarding the effect of the episodes of WPV one had on the different aspects of life. Please read the statements given below and mark the most appropriate response (based on your experience).	
B2: Personal wellbeing and self-care include activities such as sleep schedule, eating pattern, fitness, grooming, dressing etc. How much have the episodes of violence at your workplace affected your personal wellbeing and self-care?	
Not affected / mildly affected	
Moderately affected	
Severely affected	
B3: “Family life is defined as the routine interactions and activities that a family have together especially with the members who live together with parents, spouse, children.” How much has your family been affected due to the episodes of violence at your workplace?	
Not affected/ mildly affected	
Moderately affected	
Severely affected	
B4: “Social life is defined as the part of a person's time spent doing enjoyable things with others like friends, colleagues or people living in the society other than close family members.” How much has your family been affected due to the episodes of violence at your workplace?	
Not affected/ mildly affected	
Moderately affected	
Severely affected	
B5: How much do the episodes of violence at your workplace has affected your mental and psychological well-being (increased aggressiveness, irritability, low self-esteem, etc.)?	
Not affected/ mildly affected	
Moderately affected	
Severely affected	
Section C- Reporting of Incidence: This domain assesses how comfortable or confident the workers are about reporting the incidence of violence to the higher authorities.	
C1: I would be comfortable in reporting the episode of violence at my workplace to competent authorities.	
Strongly disagree	
Disagree	
Neutral	
Agree	
Strongly agree	
The statements given below (C2-C7) are some of the reasons why the incidences of violence are not reported to the authorities. Select the most appropriate choice in your opinion. To what extent do these following reasons lead to under-reporting?	
C2: Felt ashamed of reporting	
Significantly	
Somewhat significantly	
Insignificantly	
C3: A belief that no action will be taken against the perpetrator	
Significantly	
Somewhat significantly	
Insignificantly	
C4: Lack of organizational support	
Significantly	
Somewhat significantly	
Insignificantly	
C5: Lack of provision to report such incidences	
Significantly	
Somewhat significantly	
Insignificantly	
C6: The process was time-consuming	
Significantly	
Somewhat significantly	
Insignificantly	
C7: Fear that the appraisal or promotion avenues will be affected.	
Significantly	
Somewhat significantly	
Insignificantly	
Section D- Mitigation Strategies: This domain focuses on the strategies that can be useful in preventing episodes of violence at the workplace. Statements given below focus on the strategies that can be useful in preventing the episodes of violence at the workplace. Select the most appropriate choice in your opinion. To what extent do the following measures will be useful in controlling WPV in healthcare settings?	
D1: Controlling the number of attendants visiting the hospital with a patient	
Very useful	
Somewhat Useful	
Not useful	
D2: Educating patients and attendants about limitations of medical sciences and available infrastructure	
Very useful	
Somewhat Useful	
Not useful	
D3: Regular training of healthcare workers regarding soft skills (communication skills, breaking bad news, counselling skills, problem-solving skills)	
Very useful	
Somewhat Useful	
Not useful	
D4: Self-defense training of Health care workers	
Very useful	
Somewhat Useful	
Not useful	
D5:Improving healthcare facilities (like doctor-patient ratio, population-bed ratio)	
Useful	
Somewhat Useful	
Not useful	
D6: Improving facilities within a hospital (like availability of medicines and diagnostic tests)	
Useful	
Somewhat Useful	
Not useful	
D7: Improving Infrastructure facilities (like installation of CCTVs, metal detectors, alarm system)	
Very useful	
Somewhat Useful	
Not useful	
D8: Active complaint redressal system	
Very useful	
Somewhat Useful	
Not useful	
D9: Strong legislature measures like provision of significant punishment for offenders	
Very useful	
Somewhat Useful	
Not useful	
D10: Unbiased media reporting	
Very useful	
Somewhat Useful	
Not useful	
D11: Sensitizing politicians and public figures not to give immature/negative statements regarding healthcare workers	
Very useful	
Somewhat Useful	
Not useful	
Section E- Risk factors related to incidents of Workplace violence: This domain assesses the various risk factors associated with violence in healthcare settings. What is your opinion regarding the importance of the following parameters as a reason for WPV in a healthcare setting?	
E1: Unrealistic expectations of patients/attendants	
Very important	
Somewhat important	
Not important	
E2. Inappropriate knowledge about the disease/health condition	
Very important	
Somewhat important	
Not important	
E3: Poor communication skills	
Very important	
Somewhat important	
Not important	
E4: Lack of resources (equipment and medicines, doctor-patient ratio)	
Very important	
Somewhat important	
Not important	
E5: Overcrowding	
Very Important	
Somewhat Important	
Not Important	
E6: Long waiting time	
Very Important	
Somewhat Important	
Not Important	
E7: Inadequate security arrangements	
Very Important	
Somewhat Important	
Not Important	
E8: Inadequate action on receiving complaints of WPV	
Very Important	
Somewhat Important	
Not Important	
E9: Lack of respect for the authority of doctors/healthcare workers	
Very Important	
Somewhat Important	
Not Important	
E10: Negative and inappropriate media reporting	
Very Important	
Somewhat Important	
Not Important	
E11: Lack of the provision of harsh punishment for aggressors/offenders	
Very Important	
Somewhat Important	
Not Important	
E12: Lack of redressal system	
Very Important	
Somewhat Important	
Not Important	

Socio-demographic profile of the participants

Two hundred thirteen doctors and other healthcare staff working in different departments (emergency medicine, medicine, obstetrics and gynecology, and psychiatry) participated in this survey. The participants were aged 18-65 years (Mean=30.48; SD=5.08) with a slight male predominance (60.09% males). 92% of the participants were practicing in government hospitals, out of which 79% were residents of metropolitan cities. The socio-demographic details of the participants are given in Table 3.

**Table 3:** Socio-demographic profile of the participants

Characteristics	N	%
Age (in years)	30.48±5.95 (M±SD)	
Gender		
Male	128	60.09
Female	84	39.44
Prefer not to say	01	00.47
Others	00	00
Professional Qualification		
MBBS/BSc	74	34.70
MD/MSc	118	55.40
DM/PhD	21	09.86
Designation/ Job		
Consultant/faculty	34	23.94
Resident doctors	83	58.45
Nursing officer	25	17.61
Other paramedical staff	00	00
Workplace Setting		
Government hospital	194	91.08
Corporate hospital	10	04.69
Private nursing home	07	03.29
Private clinic	02	0.94
Area of working		
Metropolitan	168	78.87
Urban	42	19.72
Rural	03	01.41
The number of years of experience after completion of MBBS/BSc	6.62±5.13 (M±SD)	
Department of residency/specialization/Working		
Emergency	32	15.02
Medicine	60	28.17
Surgery	16	07.51
Obs and Gynae	39	18.31
Pediatrics	06	02.82
Trauma	14	06.57
Anesthesia and critical care	07	03.29
Others	39	18.31
Marital status		
Married	87	40.85
Unmarried	126	59.15
Others	00	00

Descriptive statistics of the survey result

It was observed that approximately 35% of the HCWs experience verbal altercations at their workplace daily, and 61% have never had any experience of physical violence. Due to episodes of violence at the workplace, 47% of the HCWS did not feel like working, which had a significant negative impact on their mental and psychological well-being. 33% of the participants did not feel comfortable reporting the incidence of violence to their authorities due to a lack of organizational support and believed no action would be taken. 

Validity of the questionnaire 

The questionnaire has good internal consistency (Cronbach’s Alpha =0.86) [15]. Multicollinearity and singularity were checked through the inter-correlation matrix. The Kaiser-Meyer-Olkin value of the questionnaire is 0.816 with a good Bartlett test of sphericity (p < 0.01), which indicates sample adequacy. The total percentage of the variance explained by the questionnaire was 67.491%, indicating good construct validity.

## Discussion

The developed questionnaire is a comprehensive and user-friendly tool with 37 items encompassing the problems related to WPV in the healthcare sector. It has five sections to assess the burden of the problem, the associated risk factors, and provide mitigation strategies to overcome it. The tool is developed on a Likert scale, which is beneficial in conducting comparative studies and can be used in different socio-cultural settings.

Section A comprises two items to assess the frequency of different types of violence experienced within healthcare settings. Apart from the prevalence, the spectrum of the various forms (verbal and physical) can also be assessed in this Section. Section B, comprising eight items, tries to analyze the impact of the episodes of WPV on the sufferers. The various components of an individual’s life such as personal (sleep schedule, eating pattern, personal hygiene), family (such as relationships with parents, spouse, children), social (friends/colleagues’/ religious practice), and psychological well-being (increased aggressiveness, low self-esteem) are found be to significantly impacted due to such incidences. It is found that despite the significant impact of these incidents on the physical and psychological well-being of the victim, such occurrences go unreported. Section C, comprising 11 items, tries to assess the reasons for not reporting these violent episodes. Mitigation strategies that can be used to mitigate the episodes of violence at the workplace are assessed in Section D, which consists of 12 items. Lastly, Section E comprises five items to highlight the various risk factors that contribute to violence in healthcare settings.

The most commonly available validated tool is made by WHO [16]. It has failed to gain popularity among researchers due to its vast and time-consuming nature. The scale is not very useful in conducting comparative studies. It fails to suggest mitigation strategies or ways to resolve the problem of WPV, which is a characteristic unique to our tool.

The developed tool has certain strengths. Firstly, it is brief and simple and may be utilized in a resource-limited setting. Secondly, it will help assess various forms of WPV, associated risk factors, and their impact. Lastly, the result of the developed tool will give government officials and healthcare practitioners in-depth knowledge regarding WPV. It will also help in devising various mitigation strategies to reduce WPV. However, there are three limitations: the semi-quantitative nature of the study, the lack of assessment of the predictive validity, and the lack of confirmatory factor analysis, which could alter the number of significant items in a construct and the total number of factors in the questionnaire.

## Conclusions

A credible and validated tool for obtaining information about workplace violence in the health sector from numerous geographic regions of the world has been established. The study is specifically looking into variables that may lead to violence and its impact, which would assist policymakers in curating various mitigation strategies to protect the victims of WPV.
